# Dr. J. W. Rhone

**Published:** 1894-08

**Authors:** 


### Obituary.

### DR. J. W. RHONE.

The subject of this sketch was born in Potter Township, Centre County, Pa., on January 20, 1833. He was sent to Pennsylvania College, Gettysburg, Pa., to complete his education, but was recalled upon the death of his father. He then, in connection with his mother, managed the ancestral farm. In 1859 he married Miss Caroline E. Keller, and engaged in mercantile business, but, finding that this occupation was neither congenial nor profitable to him, he returned for a time to his former occupation, that of a farmer. Owing to an injury, he was physically unable to endure hard labor of this character, and in the fall of 1862 he began the study of dentistry with John Wingate, D.D.S., at Bellefonte, Pa. He prosecuted his studies under his preceptor until the fall of 1863, when he entered the Pennsylvania Dental College, Philadelphia, but, owing to the necessity for active employment existing in his case, he did not graduate with his class (1865), but in 1864 began the practice of dentistry at Boalsburg, Pa. In 1874 he removed to Bellefonte, Pa., and here almost immediately his thorough knowledge of his profession and his careful and conscientious work found recognition.

In February, 1891, by a fall on an icy pavement, his right hip was broken, and he was never again able to walk without crutches; but, with characteristic determination and fortitude, he resumed operative work and continued to perform it to the day of his death. He was the father of two children,—Miss C. Ellen Rhone and Dr. Charles E. Rhone, of Bellefonte, Pa., who graduated in dentistry at the University of Pennsylvania in 1889.

Dr. Rhone in his chosen profession was thorough and progressive. He endeavored to keep abreast with the times, and, indeed, kept in advance of many of his professional brethren who had had greater advantages than he. He was respected and beloved by the entire community in which he lived his blameless life, and was the embodiment of the quiet, earnest, Christian gentleman.

He died suddenly on May 15, 1894, of organic disease of the heart, leaving a widow and the two children above named to mourn his loss.

